# Aβ degradation or cerebral perfusion? Divergent effects of multifunctional enzymes

**DOI:** 10.3389/fnagi.2014.00238

**Published:** 2014-09-11

**Authors:** J. Scott Miners, Jennifer C. Palmer, Hannah Tayler, Laura E. Palmer, Emma Ashby, Patrick G. Kehoe, Seth Love

**Affiliations:** Dementia Research Group, School of Clinical Sciences, Faculty of Medicine and Dentistry, University of BristolBristol, UK

**Keywords:** endothelin-1, angiotensin-converting enzyme, neprilysin, cerebral hypoperfusion, cerebral amyloid angiopathy, Alzheimer's disease

## Abstract

There is increasing evidence that deficient clearance of β-amyloid (Aβ) contributes to its accumulation in late-onset Alzheimer disease (AD). Several Aβ-degrading enzymes, including neprilysin (NEP), endothelin-converting enzyme (ECE), and angiotensin-converting enzyme (ACE) reduce Aβ levels and protect against cognitive impairment in mouse models of AD. In post-mortem human brain tissue we have found that the activity of these Aβ-degrading enzymes rise with age and increases still further in AD, perhaps as a physiological response that helps to minimize the build-up of Aβ. ECE-1/-2 and ACE are also rate-limiting enzymes in the production of endothelin-1 (ET-1) and angiotensin II (Ang II), two potent vasoconstrictors, increases in the levels of which are likely to contribute to reduced blood flow in AD. This review considers the possible interdependence between Aβ-degrading enzymes, ischemia and Aβ in AD: ischemia has been shown to increase Aβ production both *in vitro* and *in vivo*, whereas increased Aβ probably enhances ischemia by vasoconstriction, mediated at least in part by increased ECE and ACE activity. In contrast, NEP activity may help to maintain cerebral perfusion, by reducing the accumulation of Aβ in cerebral blood vessels and lessening its toxicity to vascular smooth muscle cells. In assessing the role of Aβ-degrading proteases in the pathogenesis of AD and, particularly, their potential as therapeutic agents, it is important to bear in mind the multifunctional nature of these enzymes and to consider their effects on other substrates and pathways.

## Introduction

The accumulation of Aβ within the brain is central to the pathogenesis of AD. The level of Aβ depends not only on its rate of production but also on the rate of its removal via various clearance pathways including enzyme-mediated degradation. Mice with inactivation of the *Mme* (neprilysin, membrane metalloendopeptidase), *Mmel1* (neprilysin-2, membrane metalloendopeptidase-like 1), *Ide*, *Ece1*, or *Ece2* gene, all have a modest (1.5–2-fold) increase in Aβ levels. Overexpression of the human orthologs of these genes in transgenic mice expressing mutant forms of human amyloid-β precursor protein (hAPP) that cause familial AD, reduce Aβ accumulation and in most, but not all cases, improves motor and cognitive performance. Findings from large post-mortem human brain studies and *in vitro* experiments have, however, revealed that the level and activity of many Aβ-degrading proteases are increased in post-mortem brain tissue and are upregulated by Aβ, suggesting that the increases are secondary to Aβ accumulation, possibly representing physiological responses to the rise in concentration of substrate. Many of the Aβ-degrading enzymes are involved in other physiological systems. For example, ECE-1/-2 and ACE are rate-limiting enzymes in the production of ET-1 and Ang II, respectively. In this review we discuss the balance between beneficial and potential deleterious effects of upregulating these vasoactive enzyme systems, and the broader relationships between Aβ and cerebral perfusion in the context of AD.

## Aβ production and clearance in alzheimer's disease

Amyloidogenic processing of APP to produce Aβ peptides results from the sequential actions of β- and γ-secretase (Selkoe, 2001; Evin and Weidemann, 2002; Mattson, 2004), yielding mostly Aβ_1−40_ and a lesser amount of Aβ_1−42_. Increased production of the less soluble Aβ_1−42_, or an increase in the ratio of Aβ_1−42:_Aβ_1−40_, is hypothesized to initiate a cascade of pathological processes leading to the development of AD (Hardy, 1997). According to the somewhat simplified view of the development of the hallmark pathological lesions of AD, extracellular Aβ_1−42_, which is more prone to aggregate (Jarrett et al., 1993) and more toxic than Aβ_1−40_, precipitates as “plaques” within the brain parenchyma and induces the development of neuritic and neurofibrillary tangle pathology, whereas much of the relatively soluble, less toxic Aβ_1−40_ reaches the cerebral blood vessels where some of it may precipitate, leading to cerebral amyloid angiopathy (CAA).

In all forms of AD, the level of Aβ in the brain is a reflection of the relative rates of Aβ production and clearance over time. In healthy individuals, the production and clearance of Aβ are rapid (estimated at ~7.6% and 8.3%, respectively, of the total volume of Aβ produced per hour (Bateman et al., 2006; Mawuenyega et al., 2010). These data suggest that even very small changes in the production or clearance of Aβ would soon cause abnormal accumulation in AD. Early-onset familial AD usually results from autosomal dominant mutations in the genes encoding APP (*APP*), presenilin-1 (*PSEN1*) or presenilin-2 (*PSEN2*) (reviewed in Tanzi and Bertram, 2005). These mutations alter the processing of APP, increasing the ratio of Aβ_1−42:_Aβ_1−40_ (Scheuner et al., 1996; Price et al., 1998). Early-onset AD can also be caused by increased production of APP, in trisomy-21 (Belza and Urich, 1986; Donahue et al., 1998), or duplication in the APP locus on chromosome 21 (Cabrejo et al., 2006; Rovelet-Lecrux et al., 2006). In sporadic AD (i.e., in over 90% of cases) there is less compelling evidence that increased amyloidogenic processing of APP is responsible for Aβ accumulation. Although studies on human post-mortem brain tissue reported increased β- and γ-secretase activities in sporadic AD (Tyler et al., 2002; Stockley et al., 2006; Miners et al., 2010a), studies in two strains of mouse transgenic for hAPP (one causing early and one causing late accumulation of Aβ) found that β-secretase activity increased only after the development of Aβ plaques (Zhao et al., 2007).

Over the last fifteen years, many studies have focused on possible abnormalities of clearance of Aβ in late-onset sporadic AD, and reported age- and disease-related deficiencies in numerous clearance pathways, including reductions in Aβ-degrading protease activities. Soluble Aβ is cleared from the brain by multiple, diverse pathways including drainage along perivascular basement membranes (Weller et al., 1998, 2000; Preston et al., 2003) and receptor-mediated transport of Aβ across the blood-brain barrier [including transport mediated by binding to low-density lipoprotein receptor-related protein (LRP-1) (Shibata et al., 2000) and through the activity of P-glycoprotein efflux pump (Lam et al., 2001; Vogelgesang et al., 2002; Kuhnke et al., 2007)]. For an in-depth review of pathways which mediate Aβ clearance, see Bell et al. (2007).

Multiple proteolytic enzymes have been identified that cleave Aβ at single or multiple sites (for comprehensive reviews, see Carson and Turner, 2002; Eckman and Eckman, 2005; Wang et al., 2006; Bell et al., 2007; Miners et al., 2011a; Nalivaeva et al., 2012). Amongst the enzymes that cleave Aβ *in vitro* are a number of zinc metalloendopeptidases including neprilysin (NEP), angiotensin-converting enzyme (ACE), and endothelin-converting enzyme-1 and -2 (ECE); thiol-metalloendopeptidases including insulin-degrading enzyme (IDE), matrix metalloproteinases [MMP-2, -9 and type-1 transmembrane MMP (MT1-MMP)]; serine proteases including myelin-basic protein (MBP), plasminogen and acyl peptide hydrolase (APEH), and cysteine proteases such as cathepsin B (for a more detailed description, refer to Miners et al., 2011a). Fragments of Aβ produced by proteolytic cleavage *in vitro* are generally considered to be less neurotoxic, and less likely to aggregate (and therefore predicted to be more easily cleared from the brain) (Mukherjee et al., 2000; Hu et al., 2001). Mice with inactivation of *Mme* (Iwata et al., 2001), *Mmel1* (Hafez et al., 2011), *Ece1, Ece2* (Eckman et al., 2003), or *Ide* (Farris et al., 2003; Miller et al., 2003) genes all have a moderate (1.5–2-fold) increase in endogenous Aβ. These KO mice did not display pathological deposition of endogenous Aβ compared to mice infused with thiorphan (Iwata et al., 2000), or phosphoramidon (Nisemblat et al., 2008), which probably reflects the overlapping substrate specificity of these inhibitors for multiple Aβ-degrading enzymes. Inactivation of NEP in hAPP mice was associated with impaired synaptic plasticity and cognitive performance (Huang et al., 2006) and was sufficient to cause plaque-like pathology (Farris et al., 2007). Conversely, overexpression of *NEP* (resulting in an 8- and 30-fold increase in protein level and enzyme activity, respectively) in hAPP transgenic (Swe/Ind) mice markedly reduced cerebral Aβ load, reversed pathological abnormalities, and improved cognitive performance and survival (Leissring et al., 2003; Poirier et al., 2006). Several other experimental strategies, aimed at increasing NEP within the CNS, by targeting either the CNS directly, or via the periphery, have suggested that upregulation of NEP might be used to treat AD (Iwata, 2003; Marr et al., 2003, 2004; El-Amouri et al., 2008; Spencer et al., 2008, 2011; Briyal et al., 2011). Induction of peripherally expressed NEP was reported to regulate Aβ level within the CNS perhaps by reducing plasma Aβ (Guan et al., 2009; Liu et al., 2009) but this, and the peripheral-sink hypothesis, has been refuted in recent studies (Walker et al., 2013; Henderson et al., 2014).

Earlier studies on human post-mortem brain tissue presented evidence to support the hypothesis that an age- and disease-related deficit in Aβ-degrading protease activity contributes to Aβ accumulation, particularly in late-onset AD. The studies reported reduced NEP and IDE mRNA levels and protein immunolabeling in AD compared to control brains (Akiyama et al., 2001; Yasojima et al., 2001; Russo et al., 2005; Miners et al., 2006; Hellstrom-Lindahl et al., 2008). However, they did not measure enzyme activity, the most relevant biological measure with respect to Aβ metabolism, were mostly based on small cohorts, in many cases did not adjust for neuronal content, and showed poor correlation between different methodologies (Russo et al., 2005; Miners et al., 2006; Hellstrom-Lindahl et al., 2008). We developed ELISA and immunocapture-based fluorogenic activity assays to measure the levels and/or specific enzyme activities of NEP, IDE, ECE-1, ECE-2, and ACE within biological tissue samples, including CSF, plasma, brain tissue homogenates and blood vessel preparations (Miners et al., 2008a,b), and used the concentration of neuron-specific enolase to adjust for neuronal content (Miners et al., 2009) in the case of NEP, IDE, ACE and ECE-2, and the concentration of factor VIII-related antigen to adjust for the endothelial cell content in the case of ECE-1 (Palmer et al., 2010). Contrary to our expectation, in a large series of well-characterized human post-mortem brains, we found that NEP, IDE, ACE, and ECE-1 activities and ECE-2 levels were all higher in AD than in age-matched controls (Miners et al., 2008c, 2009, 2010b; Palmer et al., 2009, 2010) and rose progressively with disease severity, as indicated by Braak tangle stage. The increase in the level and activity of NEP, IDE, and ACE was particularly pronounced after adjustment for neuronal loss in AD, and was directly related to the Braak tangle stage. Levels and activities of ACE and NEP were also elevated and correlated positively with Aβ_1−42_ level in Down's syndrome, which is associated with the development of AD-type neuropathology (Miners et al., 2011b). NEP, IDE, and ACE levels tended to rise with normal aging and were further increased in AD (Miners et al., 2010a). Other groups reported that other candidate Aβ-degrading proteases, including MMP-2 -3 and -9 (Yan et al., 2006; Bruno et al., 2009), cathepsin D (Gan et al., 2004; Mueller-Steiner et al., 2006; Sundelof et al., 2010) and APEH (Yamin et al., 2009), were also increased in human post-mortem brain tissue in AD, in most but not all studies (Baig et al., 2008; Barker et al., 2010). It should be noted we examined only the mid-frontal cortex, an area with a similar level of Aβ pathology to that in the temporal cortex (and more than that in the hippocampus) but affected by tau pathology only later in disease, and our approach did not take into account the relative contribution of various different cell types to overall NEP activity, an important topic for future research (see review by Saido, 2013).

Nevertheless, there is evidence to suggest that proteolysis of Aβ is not deficient in AD. On the contrary, our findings in human post-mortem tissue indicate that Aβ-degrading activity correlates positively with Aβ concentration, as would be expected for a physiological response to increasing substrate. This is supported by a large number of cell culture studies showing that exposure of neuronal, glial and vascular cell lines to Aβ (particularly fibrillar Aβ) upregulates multiple Aβ-degrading proteases including NEP, ECE, ACE, ECE-2, IDE, MMP-2 -3 and -9 (Deb et al., 1999; Jung et al., 2003; Lee et al., 2003; Leal et al., 2006; Palmer et al., 2009; Wang et al., 2009a,b; Miners et al., 2010b). A number of studies have reported a rise in Aβ-degrading protease activity in aged hAPP transgenic mice, coinciding with the appearance of parenchymal deposits of Aβ (Tucker et al., 2000; Leal et al., 2006; Yin et al., 2006). A dose-dependent increase in NEP was also observed in hAPP mice following intracerebral injection of synthetic fibrillar Aβ (Mohajeri et al., 2002, 2004). Together, these data suggest that Aβ-mediated upregulation of protease activity is secondary to Aβ accumulation in late-onset AD and that Aβ accumulates in association with a failed compensatory response rather than an overall deficiency in Aβ-degrading enzyme activity. Even if this is the case it does not preclude the potential of enhancing or administering Aβ-degrading proteases as a therapeutic target for AD. However, whilst upregulation of Aβ-degrading enzymes probably slows the accumulation of Aβ, it may also increase the cleavage of other substrates of these multifunctional enzymes. Those substrates include several peptides that play important roles in regulating cerebral blood flow.

## The endothelin system in alzheimer's disease

Endothelin-1 (ET-1) is a potent, long-lasting vasoconstrictor in the brain (Haynes and Webb, 1994). It is produced predominantly by vascular endothelial cells, but also by neurons and macrophages in human brain tissue (Palmer et al., 2012). ET-1 is generated from the inactive precursor, big-ET-1, through cleavage by ECE-1 or -2 (Yanagisawa et al., 2000). ECE-1 is expressed predominantly within the cerebral vasculature within endothelial cells (Palmer et al., 2010) but is also expressed by smooth muscle cells (Maguire, 2002), and weak neuronal and astrocytic staining of ECE-1 was observed in human brain tissue (Palmer et al., 2010). ECE-2 is expressed by pyramidal neurons, and to a lesser extent glia, within the neocortex (Palmer et al., 2009), including cells in close association with blood vessels. ET-1 has potent and sustained vasoconstrictor effects both *in vitro* and *in vivo* (Brain et al., 1988; Yanagisawa et al., 1988; Borges et al., 1989; Brain, 1989). Chronic infusion of ET-1 in to Sprague–Dawley rats led to sustained elevation in mean arterial blood pressure (Mortensen et al., 1990) and reduced perfusion, with subsequent neuronal loss (Hughes et al., 2003).

There is dysregulation of the endothelin system in AD. Several studies have shown that the concentration of ET-1 is higher in AD than controls in both the cerebral cortex (Minami et al., 1995; Palmer et al., 2012) and in cerebral blood vessels (Luo and Grammas, 2010; Palmer et al., 2013). ECE-1 and ECE-2 degrade Aβ (Eckman et al., 2001), and ECE-1 heterozygous and ECE-2 homozygous knockout mice display significantly elevated endogenous Aβ_1−40_ and Aβ_1−42_ (Eckman et al., 2001, 2003). However, we found ECE-2 level and ECE-1 activity (Palmer et al., 2009, 2010, 2013) to be higher in AD than in age-matched controls, in human post-mortem cerebral cortex and leptomeninges. We did not observe similar elevations in post-mortem tissue from patients with vascular dementia (VaD).

**Figure 1:**
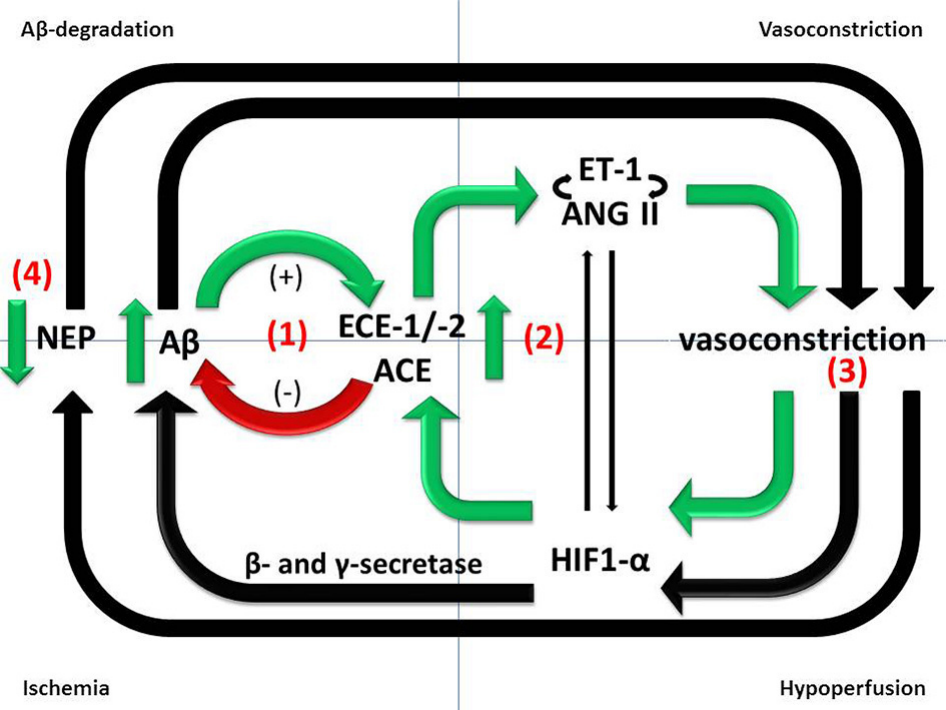
**Divergent roles of Aβ-degrading enzymes in the pathogenesis of AD**. (1) ECE-1/-2 and ACE cleave Aβ *in vitro* and ECE-1/-2 regulate endogenous Aβ level in mice. However, ECE-1/-2 and ACE activities are increased in the cerebral cortex in AD in human post-mortem brain tissue. The increases are likely to represent responses to accumulating Aβ and may mitigate the accumulation but are inadequate to prevent the level of Aβ from continuing to rise as the disease progresses. (2) Upregulation of ECE-1/2 and ACE by Aβ results in increased production of ET-1 and Ang II, both of which mediate vasoconstriction. (3) Reduced cerebral perfusion, as a result of vasoconstriction, probably increases Aβ production by promoting amyloidogenic processing of APP. (4) NEP protects vessels from Aβ-related pathology, including CAA, but NEP activity is reduced in conditions of hypoxia and oxidative stress.

Upregulation of ET-1 production in AD seems likely to be secondary to the accumulation of Aβ, since exposure of SH-SY5Y human neuroblastoma cells and human brain microvascular endothelial cells to Aβ caused upregulation of ECE-2 and ECE-1, respectively, resulting in increased production and release into the supernatant of ET-1 (Palmer et al., 2009, 2012, 2013). ET-1 production was also elevated in the cerebral vasculature of mice that had been infused with Aβ (Paris et al., 2003). The authors also showed that Aβ enhanced ET-1 induced vasoconstriction in isolated human middle cerebral and basilar arteries. Mice that overexpress hAPP showed no diminution in the contractile response of cerebral vessels to ET-1, despite an impaired response to vasodilators (Tong et al., 2005). Together, these studies suggest that upregulation of ECE-1 and -2 is a physiological feedback response to Aβ that probably increases degradation of Aβ and reduces its further accumulation but also results in increased production and release of ET-1 which mediates vasoconstriction (reviewed in Palmer and Love, 2011) (Figure 1).

It should be noted that hypoxia itself can upregulate components of the ET system. The *EDN1* gene contains a HIF1-α binding site (Takanashi et al., 2004) and ET-1 expression is induced by hypoxia or ischemia (Li et al., 1994; Yamashita et al., 2001; Ao et al., 2002; Kang et al., 2011). In another study, ECE-1 protein level and ET_A_ and ET_B_ receptor expression were increased in response to hypoxia both *in vitro* and *in vivo* (Li et al., 1994; Kang et al., 2011). Furthermore, ET-1 was shown to induce HIF1-α expression dose-dependently in cultured pulmonary smooth muscle cells (Li et al., 2012). Upregulation of more than one pathway may therefore account for increased production of ET-1 in dementia, including in AD.

ET_A_ receptor antagonists, but not dual ET_A_/ET_B_ receptor antagonists significantly reduced the impairment in learning and memory induced by Aβ in rats (Briyal et al., 2011), as well as preventing the Aβ-induced increase in expression of ET_A_ receptors and oxidative stress. Additionally, an ET_B_ receptor agonist elevated CBF in normal rats and acted as an anti-apoptotic factor, counteracting the neurotoxicity and cognitive impairment induced by Aβ (Briyal et al., 2014). The findings suggest that ET_A_ receptor antagonists, which are already used in the treatment of pulmonary hypertension (Palmer and Love, 2011), and potentially ET_B_ receptor agonists, have therapeutic potential in AD.

## The renin-angiotensin system in alzheimer's disease

The renin-angiotensin system (RAS) plays an important role in fluid homeostasis and regulation of blood pressure. ACE catalyzes the conversion of angiotensin I (non-vasoactive) to the vasoconstrictor, angiotensin II. ACE also cleaves and thereby inactivates the vasodilator, bradykinin. These vasopressor activities can be blocked either by ACE-inhibitors, a standard treatment for hypertension or by Ang II receptor antagonists that inhibit Ang II-mediated vasopressor effects. ACE is expressed by the endothelium and neurons within the brain (Turner and Hooper, 2002; Miners et al., 2008c).

The involvement of the RAS, particularly ACE, in the pathogenesis of AD has been studied extensively (reviewed in Kehoe et al., 2009). ACE cleaves Aβ *in vitro* (Hu et al., 2001; Hemming and Selkoe, 2005; Oba et al., 2005; Sun et al., 2008; Toropygin et al., 2008) and retards Aβ aggregation and neurotoxicity (Hu et al., 2001). A recent study indicated that ACE-mediated cleavage of Aβ_1−42_ resulted in the formation of the less neurotoxic Aβ_1−40_ species (Zou et al., 2007). The exact contribution of ACE to the regulation of Aβ *in vivo* remains unclear (reviewed in Kehoe et al., 2009). Eckman et al. (2006) reported that endogenous Aβ levels were unchanged in an *ACE* knockout mouse and in mice treated with ACE inhibitors. In support of the earlier studies, Hemming et al. (2007) found that captopril did not significantly affect Aβ levels in two human APP transgenic mouse lines. More recently, however, Wang et al. (Zou et al., 2007) showed that administration of captopril for 7 or 11 months to 6-month old tg2576 mice (transgenic for the Swedish double mutation in human APP) promoted Aβ deposition. The divergence of results could reflect a difference in the species and age of the animals used, and the respective dosage regimes, and highlights the need for further investigation.

ACE level and enzyme activity are increased within the cerebral cortex in AD. This has been demonstrated in multiple human post-mortem studies (Arregui et al., 1982; Barnes et al., 1991; He et al., 2006; Miners et al., 2008a). We showed a strong correlation between ACE activity and disease severity, as indicated by Braak tangle stage and parenchymal Aβ load (Miners et al., 2008a). Thus, ACE-mediated cleavage of Aβ is not impaired in AD. We also showed that ACE protein level and activity were upregulated in human neuronal SH-SY5Y cells in response to exposure to synthetic, aggregated Aβ_1−42_ (Miners et al., 2008a) suggesting that induction of ACE is a likely to be a physiological response to increasing Aβ level and may help to prevent further Aβ accumulation.

A common Alu (indel) insertion(I)/deletion(D) polymorphism (rs 1799752) in intron 16 of the *ACE* gene was reported to be associated with sporadic AD, the greatest risk being associated with homozygosity of the I allele (Kehoe et al., 1999). This was confirmed in several meta-analyses (Narain et al., 2000; Kehoe et al., 2003; Elkins et al., 2004; Lehmann et al., 2005) and was supported to some extent by some of the earlier whole-genome association studies (Li et al., 2008; Thornton-Wells et al., 2008) although a number of more recent and larger studies have failed to find any association. In the original studies, homozygosity of the D and I alleles was associated with the highest and lowest ACE protein level in the periphery, respectively, (Rigat et al., 1990) although this association only partly explains the total variance of ACE in the periphery (Terao et al., 2013). We found that the *ACE* II genotype was associated with reduced ACE protein level in the CSF (Miners et al., 2010b). Other studies showed significant association between *ACE* variants and Aβ_1−42_ concentration or the Aβ_1−42_:Aβ_1−40_ ratio in the CSF (Lehmann et al., 2005; Kauwe et al., 2009). These studies suggested that an association between the *ACE* polymorphism and AD might be mediated through reduced ACE-mediated proteolysis of Aβ. However, on analysis of post-mortem brain tissue, we subsequently found that the putative AD risk genotype, *ACE* II, was associated with increased rather than decreased ACE activity (Miners et al., 2010b). ACE expression within the brain did not mirror findings in the plasma and CSF; this finding is in keeping with other evidence that RAS within the brain functions in isolation from the periphery. In addition, ACE enzyme activity did not correlate with ACE protein level, possibly due to the effects of post-translational modification on ACE enzyme activity.

Although it now seems unlikely that ACE contributes substantially to the degradation of Aβ *in vivo*, there is evidence that the RAS influences several other processes that contribute to the development of AD. Intracerebral infusion of Ang II in male Sprague–Dawley rats resulted in significant cognitive impairment, increased amyloid pathology [due to elevated β- and γ-secretase activity (Zhu et al., 2011)] and an increased level of phosphorylated tau (Tian et al., 2012). Ang II infusion also resulted in stimulation of type 1 Ang II receptors (AT1Rs) that mediate vasoconstriction. These effects were prevented by losartan, an AT1R receptor antagonist. Additionally, Ang II inhibits potassium-mediated release of ACh (Barnes et al., 1992), and influences tumor necrosis factor-α and transforming growth factor β signaling (Hamdi and Castellon, 2004) and blood-brain barrier maintenance (Wosik et al., 2007). Administration of the Ang II receptor antagonist valsartan in tg2576 mice (transgenic for the Swedish double mutation in human APP) resulted in lower Aβ deposition and improved cognitive performance (Wang et al., 2007). Danielyan and colleagues similarly showed that intra-nasal administration of losartan significantly reduced Aβ pathology (Danielyan et al., 2010). In view of these pleotropic actions of Ang II, that are associated with a number of AD pathological processes, and the recent observations in animal models of AD treated with losartan, AT1Rs have come under scrutiny as a potential therapeutic option for AD. Data from large retrospective clinical studies (Li et al., 2010; Davies et al., 2011) and secondary explorations of clinical trial studies (Gao et al., 2013; O'Caoimh et al., 2014) suggest that use of ARBs may be protective against the development and progression of AD (reviewed in Ashby and Kehoe, 2013). The finding in some studies that the reduction in risk is associated with ARBs but not (or not to the same extent) with ACE inhibitors (Kehoe et al., 2013) may reflect a beneficial effect of maintaining ACE-mediated Aβ degradation. Clinical trials are underway to determine whether losartan (ISRCTN93682878) or perindopril vs. telmisartan (NCT02085265) can reduce AD-associated pathology and cognitive decline in mild to moderate AD.

Chronic exposure to elevated ACE activity may increase the risk of dementia by promoting cerebrovascular disease. In a large prospective cohort of patients (the SMART-MR study) with symptomatic atherosclerotic disease, elevated serum ACE activity was associated with increased risk of ischemic stroke and high blood pressure (Jochemsen et al., 2012a) and increased progression of white matter ischemic damage (Jochemsen et al., 2012b). ACE expression is directly regulated by hypoxia via a HIF1-α-dependent mechanism. Krick et al. (2005) observed increased ACE expression in cultured human FB_PA_ (advential fibroblasts) that was dependent on HIF1-α expression. Upregulation of more than one pathway may therefore account for increased production of Ang II in dementia, including AD.

Emerging studies highlight possible interactions between the ET-1 and RAS in AD. Ang II interacts with the ET system to increase ET-1 production from endothelial cells (Dohi et al., 1992) and vascular adventitial fibroblasts (An et al., 2006) via a mechanism involving NADPH oxidase (An et al., 2007). Ang II mediates the transcriptional regulation of the preproenothelin-1 (*ppET-1*) gene and induces the synthesis of ET-1 *in vitro* (Rossi et al., 1999). Ang II was also shown to increase tissue ET-1 *in vivo* (Moreau et al., 1997). In addition, ET-1 also contributes to the vascular actions of Ang II (Balakrishnan et al., 1996; Moreau et al., 1997) and ET-1 enhances the conversion of Ang I to Ang II in pulmonary artery endothelial cells (Kawaguchi et al., 1991).

## Hypoperfusion and vascular dysfunction in alzheimer's disease

There is increasing evidence that vascular dysfunction, reduced cerebral blood flow and AD are interdependent pathological processes. In large epidemiological studies, cardiovascular risk factors (diabetes, hypertension, high cholesterol levels, atherosclerosis, obesity) have emerged as risk factors for AD (DeKosky et al., 1990; Johnson et al., 2005; Ruitenberg et al., 2005; Mak et al., 2012). Brain ischemia and stroke were reported to increase the risk of dementia and AD (de la Torre, 2005; Dickstein et al., 2010). Many AD patients have some degree of cerebrovascular pathology including white matter ischemic lesions (Brun and Englund, 1986a,b; Englund et al., 1988; Englund and Brun, 1990; Ellis et al., 1996; Kalaria, 2000) and most AD patients have CAA (Esiri and Wilcock, 1986; Love et al., 2003; Jellinger and Attems, 2006). Brain ischemia is the defining pathogenic process in most patients with VaD but in AD cerebral blood flow is also usually reduced (Sharp et al., 1986; Jagust et al., 1987; Schuff et al., 2009) and contributes to cognitive impairment. Reduced cerebral blood flow probably anticipates the development of dementia in AD (Ruitenberg et al., 2005), and occurs well before any pathological or neurological abnormalities in mouse models of AD (Niwa et al., 2002a,b; Iadecola, 2004).

There is evidence from some although not all studies (Chui et al., 2012) that ischemia is not only an additive cause of brain damage in AD but also contributes to the neurodegenerative disease processes. Transient brain ischemia resulting from cardiac arrest was shown to cause a marked increase in serum Aβ_1−42_ in humans (Zetterberg et al., 2011). Ischemia, or its *in vitro* simulation by combined deprivation of oxygen and glucose, is associated with increased production of Aβ in animal and cell culture models of AD (Zhang et al., 2007; Guglielmotto et al., 2009; Zhiyou et al., 2009). This probably reflects increased amyloidogenic processing of APP by γ- and β-secretase in response to hypoxia (Sun et al., 2006; Li et al., 2009), resulting in increased plaque formation (Garcia-Alloza et al., 2011). HIF-1α is the primary sensor of tissue hypoxia (Wenger, 2002). HIF-1α subunits are stabilized under conditions of hypoxia, dimerize with HIF-1β and translocate to the nucleus, where they associate with hypoxia-response elements and bind transcriptional co-activators to induce the expression of hypoxia-responsive genes. It is likely that Aβ also contributes indirectly to the reduction in CBF, by mediating vasoconstriction (Claassen and Zhang, 2011) (Figure 1).

We have reviewed (above) evidence that Aβ upregulates ET-1 and Ang II production. In addition, Aβ peptides were reported to enhance the vasoconstrictor action of a thromboxane analog (Niwa et al., 2001; Iadecola, 2003). In mouse models of AD, Aβ impaired cerebral autoregulation and functional hyperemia (Niwa et al., 2000, 2001, 2002a), although in studies to date, cerebral autoregulation has not been shown to be impaired in AD (Claassen and Zhang, 2011). The Iadecola group showed that oxidative stress plays a critical role in the attenuation of functional hyperemia by Aβ_1−40_ (Park et al., 2004). The *in vitro* finding by Palmer et al. (2013) that an Aβ_1−40_ induced increase in ET-1 production by endothelial cells could be blocked by co-administration of the antioxidant superoxide dismutase suggests that the attenuation of functional hyperemia by oxidative stress may be partly mediated by ET-1. Cerebral ischemia also induced tau phosphorylation, leading to the death of basal forebrain cholinergic neurons which are profoundly depleted in AD (Zheng et al., 2002; Li et al., 2011). Li et al. (2011) reported that ET-1 exacerbated Aβ deposition, tau phosphorylation and cognitive impairment after intracerebral injection of Aβ in rats.

## Post-mortem assessment of cerebral hypoperfusion

Evaluation of the possible contribution of ischemia to the development or progression of AD and other neurological disorders has been impaired by the lack of objective, quantifiable markers of hypoperfusion in post-mortem brain tissue. Pathologists have relied on morphological assessment of paraffin sections, and although this approach has been informative it is limited by its subjectivity and relative insensitivity, relying on the identification of infarcts and regions of white matter rarefaction rather than more subtle manifestations of ischemia. One potential approach to quantifying ante-mortem hypoperfusion by examination of post-mortem brain tissue is the measurement of gene transcripts and proteins involved in the molecular responses to brain ischemia. As noted above, HIF-1α subunits are the principal sensors of tissue hypoxia (Acker and Acker, 2004). Under conditions of chronic or intermittent hypoxia there is an increase in HIF-1α transcription (Nanduri et al., 2008; Powell, 2009). Tissue hypoxia also causes upregulation of a large number of other genes. Some, like VEGF and heme-oxygenase (HO-1), are upregulated by HIFs (Liu et al., 1995; Shweiki et al., 1995; Pham et al., 2002); others, such as neuroglobin are upregulated independently of HIFs (Sun et al., 2001). Fernando et al. (2006) reported elevated immunolabeling of astrocytes or microglia for HIF-1α and HIF-2α within deep white matter lesions in the elderly. We measured VEGF by ELISA, in both cerebral cortex and white matter (Barker et al., 2013) from human post-mortem brains, and found it to be elevated in the cerebral cortex in AD independently of the severity of small vessel disease or CAA. VEGF was not elevated in the white matter in AD (Barker et al., 2014).

In a study of two independent post-mortem cohorts, we recently showed that the ratio of myelin-associated glycoprotein (MAG) to proteolipid protein 1 (PLP1) in the white matter correlated inversely with the severity of small vessel disease, and so presumably with the degree of ante-mortem ischemia (Barker et al., 2013). Our rationale for measuring this combination of myelin proteins was that the transport of myelin proteins from the oligodendrocyte cell body where they are synthesized, to their site of insertion in the myelin sheath, is an energy-dependent process and MAG, being expressed only adaxonally, far from the oligodendrocyte cell body, is more susceptible to ischemia than is PLP, expressed throughout the myelin sheath. We have found that MAG is also sensitive to ischemia in the gray matter and are currently using a combination of methods to assess the relationships between the activity of the endothelin and RAS, cerebral hypoperfusion, neurodegenerative pathology and white matter ischemic damage in AD and other dementias.

**Figure 2:**
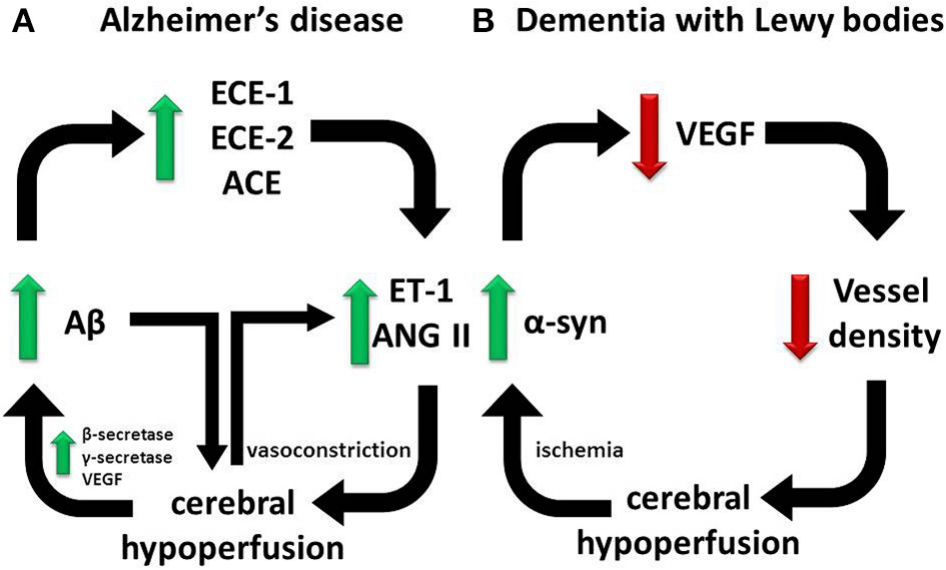
**Divergent pathways responsible for cerebral hypoperfusion in dementia**. Our observations on human post-mortem brain tissue suggest that **(A)** in AD, cerebral hypoperfusion is mediated in part by elevated ECE-1/-2 and ACE in the cerebral cortex in AD in response to accumulating Aβ, whereas **(B)** in DLB, cerebral hypoperfusion is mediated by reduced vessel density in association with a reduction in VEGF expression, possibly in response to α-syn accumulation mediated by ischemia. In each pathway a vicious cycle ensues that accelerates disease progression.

Our findings to date point toward different processes underlying ischemic changes in the cerebral cortex and white matter in AD, VaD and dementia with Lewy bodies (DLB) (Figure 2). Whereas ET-1 production is significantly increased in the cerebral cortex in AD, presumably reflecting locally elevated Aβ and oxidative stress, it is reduced in the white matter, as would be expected for a physiological adaptation to reduced blood flow. It should be noted that multiple, topographically remote processes are likely to contribute to white matter ischemia in AD. These include meningeal and cortical CAA, Ang II- and ET1-mediated constriction of perforating arterioles that traverse the cortex to supply the white matter, and in some patients, systemic processes such as postural hypotension (Ballard et al., 2000) and atrial fibrillation (Ott et al., 1997).

There is little published information on the possible contribution of cerebral hypoperfusion to other dementias. Patients who have DLB are particularly prone to neurocardiovascular instability (Ballard et al., 1998), which correlates with the severity of white matter ischemic lesions detectable by MRI (Kenny et al., 2004). Several other studies have demonstrated reduced perfusion of the parieto-occpital cortex in DLB (Ishii et al., 1999; Lobotesis et al., 2001; Colloby et al., 2002; Firbank et al., 2003; Fong et al., 2011). We recently reported that hypoperfusion in the occipital cortex of patients with DLB was associated with reduced microvessel density (as determined by measuring the level of factor VIII-related antigen) (Barker et al., 2014) and a reduction in VEGF (Miners et al., 2014). The VEGF level in DLB correlated positively with the ratio of MAG:PLP, in keeping with a direct relationship between reduced VEGF, reduced vascularity and hypoperfusion (leading to reduced MAG:PLP). The mechanism of VEGF reduction in the occipital cortex in DLB is unclear. However, it seems to be related to the underlying disease process, correlating negatively with the levels of total α-syn and of α-syn phosphorylated at serine-129 (Figure 2). VEGF level was reduced in SHSY-5Y cells overexpressing human α-syn. Conversely, oxygen-glucose deprivation increased α-syn level and aggregation. In contrast to AD, in DLB the level of ET-1 and activity of ACE were similar to levels in age-matched controls.

## Neprilysin and cerebral amyloid angiopathy

The most common form of CAA is caused by cerebrovascular accumulation of Aβ in the walls of leptomeningeal and cerebrocortical blood vessels, particularly arterioles (Revesz et al., 2002, 2003). CAA increases in prevalence with age, to about 45% in those over 80 years, and is present in over 90% of patients with AD (Love et al., 2003). Aβ_1−40_ is the most abundant isoform in vessel walls (Gravina et al., 1995). It is thought to be predominantly, if not exclusively, of neuronal origin (Calhoun et al., 1999; Burgermeister et al., 2000; van Dorpe et al., 2000). The Aβ accumulates initially in the basement membrane but eventually causes the death of the cerebrovascular smooth muscle cells (CVSMCs) and replaces the tunica media. Particularly in larger vessels, Aβ may also deposit in the adventitia (Weller et al., 2009a). Cerebrovascular accumulation of Aβ may be caused by increased production of this peptide, in familial forms of AD and CAA. However, in most sporadic cases, decreased removal, particularly as a result of the effects of age on vessel walls, is likely to be the major cause of CAA. Arteriosclerosis and age-related changes to the composition of the arterial basement membranes may impede the perivascular drainage of Aβ, as does preexisting CAA (Weller et al., 2009b; Hawkes et al., 2011).

Another determinant of the likelihood of vascular accumulation of Aβ is the activity of Aβ-degrading proteases within the tunica media. We and others showed that NEP is abundantly expressed by smooth muscle cells within larger arterioles in the cerebral cortex and leptomeninges (Carpentier et al., 2002; Miners et al., 2006) in human brain tissue, and that the level of NEP is reduced in AD patients with severe CAA. The reduction is not simply a consequence of the loss of smooth muscle cells. We demonstrated that the reduction of NEP protein in CAA affected both Aβ-laden and non-Aβ-laden vessels (Miners et al., 2006). We also showed that NEP enzyme activity is reduced in CAA, in both the leptomeninges (within which the only source of NEP is the smooth muscle cells in the blood vessels) and vessel-enriched preparations of cerebral cortex (Miners et al., 2011c). The reduction in NEP activity was still evident after adjustment for the smooth muscle actin content of the samples and was therefore not simply a consequence of death of CVSMCs, although loss of CVSMCs, whether from arteriosclerosis or as a consequence of CAA, would be expected to exacerbate any preexisting deficiency in vessel-associated NEP.

To model the role of NEP in CAA we manipulated NEP activity in cultured human brain-derived vascular smooth muscle cells, using siRNA knock-down of NEP, to reduce activity, or transfection of NEP cDNA to enhance activity, and measured cell death after adding recombinant Aβ_1−42_ (Miners et al., 2011c). We found that NEP protected against Aβ_1−42_-mediated cell death. We also analyzed the influence of *APOE* genotype on vessel-associated NEP activity. The relationship between *APOE* and vessel-associated NEP activity mirrored that between *APOE* genotype and risk of AD and CAA: after adjustment for smooth muscle actin, NEP activity was highest in blood vessels from patients with *APOE* ε2/3 genotype and decreased stepwise through *APOE* ε3/3, ε3/4, and ε4/4 genotypes. The possible contribution to CAA of changes in the activity of other vessel-associated Aβ-degrading enzymes, including ECE-1 and IDE (Morelli et al., 2004; Lynch et al., 2006) have yet to be assessed.

## Concluding remarks

Multiple Aβ-degrading proteases are upregulated in AD in response to the rising concentration of Aβ. This upregulation probably mitigates the accumulation and toxicity of Aβ, including in the context of CAA, but also enhances the degradation of other substrates of these proteases. Those substrates include the vasodilator bradykinin, and precursors of the potent vasoconstrictors ET-1 and Ang II. The resulting vasoconstriction is likely to contribute to reduced cerebral blood flow in AD and perhaps also to the neurodegenerative disease process itself, through increased production, accumulation and deposition of Aβ and increased phosphorylation of tau. Reduced cerebral blood flow also contributes to VaD and dementia with Lewy body diseases but the distribution and mechanisms of hypoperfusion are different from those in AD. Our findings underline the limitations of highly reductionist approaches for assessing the actions of multifunctional proteases, and the importance of validating experimental findings by analysis of human brain tissue.

### Conflict of interest statement

The authors declare that the research was conducted in the absence of any commercial or financial relationships that could be construed as a potential conflict of interest.
